# Provider Perspectives on Sleep as a Determinant of Health and Housing Outcomes among Veterans Experiencing Homelessness: An Exploratory, Social-Ecological Study

**DOI:** 10.3390/ijerph20095739

**Published:** 2023-05-08

**Authors:** Elizabeth M. Moore, Lillian Gelberg, Michael Soh, Cathy Alessi, Roya Ijadi-Maghsoodi

**Affiliations:** 1 Department of Psychiatry, University of Pennsylvania, Philadelphia, PA 19104, USA; 2 Office of Healthcare Transformation and Innovation, VA Greater Los Angeles Healthcare System, Los Angeles, CA 90073, USA; 3 Department of Family Medicine, David Geffen School of Medicine at UCLA, Los Angeles, CA 90095, USA; 4 Department of Health Policy and Management, UCLA Fielding School of Public Health, Los Angeles, CA 90095, USA; 5 Center for Health Professions Education, Uniformed Services University of the Health Sciences, Bethesda, MD 20814, USA; 6 Geriatric Research, Education and Clinical Center, VA Greater Los Angeles Healthcare System, Los Angeles, CA 90073, USA; 7 Department of Psychiatry and Biobehavioral Sciences, David Geffen School of Medicine at UCLA, Los Angeles, CA 90095, USA; 8 VA Health Service Research & Development (HSR&D), VA Greater Los Angeles Healthcare System, Los Angeles, CA 90073, USA; 9 UCLA Division of Population Behavioral Health, Jane and Terry Semel Institute for Neuroscience and Human Behavior at UCLA, Los Angeles, CA 90024, USA

**Keywords:** veterans, sleep, homeless

## Abstract

Sleep problems are common among United States (U.S.) veterans and are associated with poor health, mental health, and functioning. Yet, little is known about insufficient sleep and factors contributing to sleep disparities among veterans experiencing homelessness. We conducted semi-structured interviews to better understand the clinical, environmental, and structural factors contributing to insufficient sleep among veterans and to improve care for this population. Interviews were conducted with 13 providers caring for veterans experiencing homelessness, including physicians, psychologists, nurses, social workers, and peer support specialists. Providers worked at a West Coast VA institution serving a large population of veterans experiencing homelessness. Interviews were analyzed for themes pertaining to sleep using the social-ecological model as a framework. On an individual level, factors influencing sleep included psychiatric disorders and use of substances. On an interpersonal level, factors included safety concerns while sleeping. On an environmental level, factors included noise and proximity to others as barriers to sleep. On the organizational level, logistical issues scheduling sleep clinic appointments and lack of transportation to attend sleep clinic appointments were identified as treatment barriers. These findings can inform future research studying the impact of sleep on health and housing outcomes and interventions addressing sleep among veterans experiencing homelessness.

## 1. Introduction

Insufficient sleep—meaning less than seven hours of sleep per night—is a public health problem [1], with chronic insomnia and obstructive sleep apnea (OSA) the most common sleep disorders seen in the primary care setting [2]. Insufficient sleep is also highly prevalent in outpatient psychiatric settings [3] and puts patients at risk for worsening of mental health conditions [4]. The adverse health effects of poor sleep include impaired immune function [5], worsened quality of life, and increased mortality [6]. Further, there is increasing research that under-resourced and minoritized populations experience significant sleep health disparities—including insufficient and poor-quality sleep—due to structural and environmental factors [7]. Researchers have called to urgently address sleep health disparities among under-resourced populations through structural and culturally sensitive intervention approaches, especially during the COVID-19 pandemic where poor sleep may exacerbate poor health outcomes [8].

The impact of insufficient sleep in the homeless population is poorly understood. People experiencing homelessness are highly vulnerable to negative health outcomes, including elevated rates of poor health, mental illness, and poor social connection [9,10,11,12]. The limited available evidence suggests that people experiencing homelessness have high rates of sleep disturbance. A survey of people experiencing homelessness in France reported fewer hours of sleep, higher rates of insomnia, and greater use of sleep medications than the general population [13]. A study of people experiencing homelessness in Canada found that 38% reported sleep difficulty [14], and a United States (U.S.)-based study found that 44% of homeless-experienced adults reported sleeping six or fewer hours per night [15], less than the recommended minimum of seven hours [16]. Additionally, a study of homeless-experienced women found that most self-reported restless sleep [17].

The causes of insufficient sleep among people experiencing homelessness have not been well-defined in research studies but are likely multifactorial. People experiencing homelessness may have poor access to healthcare including sleep disorder treatment [18]. People sleeping outside may be susceptible to local policies that may impair their ability to safely sleep. For example, in Los Angeles—a city experiencing a homelessness crisis—individuals sleeping outside must dissemble their tents by 6:00 A.M., despite that individuals experiencing homelessness often choose to sleep during the day [19]. 

There is a particular dearth of research about sleep disturbance among military veterans who have experiences with homelessness (hereafter “homeless-experienced” veterans), despite that insufficient sleep is increasing among the veteran population [20]. Homeless-experienced veterans are a particular group that may be especially vulnerable to sleep disturbance and suffer heightened sequelae due to high rates of mental illnesses, including combat-related post-traumatic stress disorder (PTSD) and substance use disorders [21,22]. Despite Department of Veteran Affairs (VA) efforts to address the social determinants of health among veterans, including addressing homelessness through various homeless programs [23,24,25,26], very little is known about sleep as a determinant of health among homeless-experienced veterans. In addition to a limited body of literature on sleep disparities among veterans experiencing homelessness, the existing research on sleep and homelessness does not address the relationship between sleep and important outcomes such as housing retention. 

To further understand the issue of insufficient sleep among homeless-experienced veterans and the contributing clinical, environmental, and structural factors related to sleep disparities among this population, we conducted qualitative interviews with homeless services providers and organized our findings into a social-ecological framework. As our purpose was to understand the topic of sleep disparities among homeless-experienced veterans to inform future research and care, this was an exploratory qualitative study. As a result of these findings, we (1) clarify gaps in research around sleep among homeless-experienced veterans, (2) provide a rubric for identifying sleep concerns in the clinical setting that is attuned to the needs and barriers to quality sleep of homeless-experienced veterans through the social-ecological model, and (3) identify policy and community-based strategies to improve care and reduce sleep disparities for this population. Given mounting evidence of the role of sleep disparities among under-resourced and minoritized populations, we hope that a better understanding of sleep within the homeless-experienced veteran population will clarify the relationships between sleep and housing retention, chronic disease, and well-being for this important population. 

## 2. Materials and Methods

### 2.1. Participants

We conducted semi-structured interviews between April 2018 and June 2019 with providers with expertise in caring for homeless-experienced veterans. We recruited providers from a multidisciplinary primary care clinic for homeless-experienced veterans and from social service programs and permanent supportive housing programs within one large U.S. VA healthcare system, serving the largest number of homeless veterans in the country [27].

We purposively recruited providers from multiple disciplines, including across primary care, mental health, housing services, social services, and peer support services. Providers were identified by clinic and social service leaders and invited to partake in interviews via email invites. Twenty-four providers were contacted, and thirteen volunteered to complete a full interview. Eligibility criteria were serving as a provider for homeless-experienced veterans and being able to take part in an interview. We define providers to include clinical providers, social service providers, and peer support specialists. Although all providers interviewed provided care to homeless-experienced veterans, their roles and interactions with veterans were quite varied, for example, from clinicians providing health and mental healthcare, to social services, to peer supports who often work closely with veterans outside the clinic setting and provide services such as peer mentorship, support, and care linkage. Peer specialists are VA employees who are engaged in their own recovery, often have a history of homelessness, and who help other veterans engage with mental health, substance use, and social services [28]. The Institutional Review Board (IRB) reviewed all study materials and instruments and formally designated the activity as quality improvement.

### 2.2. Procedures

Semi-structured interviews lasting 60 min were conducted by members of the research team trained in conducting interviews, in English either over the phone or in a private setting on the health center campus. No one else was present. Most interviews were conducted by two team members (LG and CA); one interview was conducted by two other team members (EM and MS). 

The semi-structured interview guide consisted of questions inquiring about (1) provider role, (2) sleep problems among homeless-experienced veterans, (3) factors contributing to sleep problems, (4) sleep problems and comorbidities, (5) treatment of sleep problems, and (6) barriers to treatment. Detailed notes were taken during interviews by both interviewers. Interviews were completed when thematic saturation was reached [29]. Participation was voluntary, and incentives were not provided. No repeat interviews were given, and participants did not ask to review transcripts. 

### 2.3. Analysis

The interview notes were initially reviewed independently by three members of the research team trained in qualitative research (EM, MS, RIM). For each participant, notes from both interviewers were reviewed, with the set of notes with the most detail and richness selected for coding. Preliminary themes based on the interview guide were first discussed. The same three members of the research team then met and iteratively discussed, collapsed, and created codes, developing a mutually agreeable codebook informed by the social-ecological model [30] (as described below). The codebook categories focused on sleep among homeless-experienced veterans including sleep problems; related mental health, substance, and health comorbidities; safety; the environment related to sleep; sleep treatment; and logistical barriers. Two team members (EM and MS) coded the interview notes to reduce risk of interpretive bias [31]. All discrepancies in coding were resolved in discussion with the qualitative team leader (RIM). Codes were inputted into Microsoft Excel [32], which allowed for better visualization and interpretation of codes and themes. Glaser and Strauss’ constant comparative method was utilized to identify main themes from the interviews [33].

## 3. Results

**Figure 1:**
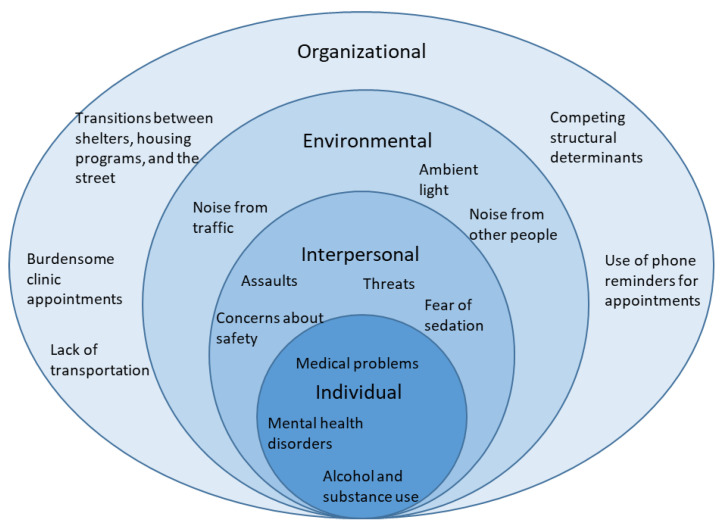
Social-ecological model of barriers to sleep among homeless-experienced veterans described by providers. The different shades of blue represent the different levels of the social-ecological model, with the darkest shade representing the individual and the lightest shade representing the complex organizational groupings of people and places.

Participants (N = 13) included three primary care clinicians (two nurse practitioners and one physician), four mental health clinicians, one registered nurse, one social worker, and four peer support specialists. Interview themes were framed using the social-ecological model [30], consisting of four levels: individual, interpersonal, environmental, and organizational (see Figure 1). For this study, individual factors refer to personal characteristics or actions of veterans; interpersonal factors involve interactions between veterans and their peers; environmental factors relate to characteristics of the sleep environment; and organizational factors are practices and policies within social services and healthcare. Below, factors related to poor sleep among homeless-experienced veterans are described across the four levels of the social-ecological model and also depicted in Figure 1. 

### 3.1. Individual Factors

#### Mental Health, Substance Use, and Medical Problems

All of the providers interviewed brought up that poor sleep was an issue among homeless-experienced veterans. Providers highlighted that an entanglement of mental health, substance use, and medical problems afflicted veteran patients and impacted their sleep. First, most providers discussed that mental health problems were linked to poor sleep among their patients. Although all providers brought up the issue of mental health impacting sleep, mental health providers noted that trauma histories are common among homeless-experienced veterans, and most connected PTSD symptoms of hypervigilance and nightmares with poor sleep in their patients. The majority of providers, spanning the different disciplines, also identified substance use as a factor of poor sleep in this population. Providers described examples where veterans utilized substances to address sleep barriers. For example, one provider described how patients would use methamphetamines to prevent falling asleep to avoid nightmares (Provider 8). Several providers noted that other substances, particularly heroin, were also used in this population to help induce sleep. Many of the providers interviewed—including primary care clinicians, mental health clinicians, and peer specialists—noted the disruptive impact of alcohol on sleep among their patients. Finally, some providers described that once housed, veterans continued to be plagued by mental health and sleep issues despite obtaining housing. 

With regard to medical problems, several primary care providers brought up that medical problems, such as pain and nocturia, could contribute to poor sleep. For example, a primary care provider described veterans sleeping in their cars and experiencing worsened aches and pains (Provider 11). Although this topic was touched upon by primary care providers, the issue of medical problems contributing to sleep was not raised among mental health, social work, or peer support providers. 

### 3.2. Interpersonal Factors

#### Safety

Concern about safety while homeless was identified as a major factor for disrupted sleep by providers. This issue was mentioned by most providers interviewed, including mental health, primary care, and peer specialist providers. Providers conveyed that veterans might avoid sleeping at night due to fear of theft or violence. A social worker noted that many of her patients would stay up all night to protect themselves and their belongings (Provider 4). A mental health clinician reasoned that individuals who feel unsafe may have trouble achieving deep sleep and may awaken easily (Provider 1). 

Several providers voiced that the feeling of being unsafe can often persist when veterans receive permanent housing. Even when housed through permanent supportive housing programs, which enable independent living through a combination of housing assistance and voluntary support services, a primary care clinician observed that veterans may feel safe only after a year in their new homes (Provider 7). 

### 3.3. Environmental Factors

#### 3.3.1. Sleeping Space

Nearly every provider commented that the myriad environmental conditions that homeless-experienced veterans are exposed to can disrupt sleep. For example, a nurse listed environmental issues on the street, including heat and noise from traffic and other people, that could disrupt sleep among those living outside (Provider 13). 

Several providers commented on the disruptive environmental conditions in shelters. Emergency shelters typically offer temporary beds, the duration of stay in the shelters is often capped between three and 60 nights, and shelters are dormitory-style settings where clients share rooms. Providers expressed that the noise from other clients, such as snoring, could disrupt sleep. Two mental health providers brought up that inflexible shelter rules would negatively impact sleep, explaining that, for instance, shelters might require clients to be inside the shelter by 10:00 p.m. and out of bed by 6:00 a.m. Providers noted that for those who work night shifts, which they described as common among their patients, these rules preclude adequate sleep (Provider 10, Provider 12). 

Transitional housing—which typically provides individual rooms or apartments for stays of three months to three years and includes supportive services [34]—was also described as an environment with sleep barriers to veteran patients. A primary care provider noted that noise within facilities, as well as the potential for fights among residents, may keep veterans awake (Provider 5). A nurse expanded upon this, describing walls that reverberate sound and sleeping quarters that are close to noisy showers as potential sleep disturbances within housing facilities. This provider explained that veterans might find it difficult to build healthy sleep patterns in this environment (Provider 13). 

#### 3.3.2. Interference with Treatment

Providers noted that suboptimal environments can render management of sleep problems difficult. For example, cognitive behavioral therapy for insomnia (CBT-I) is a first-line treatment for chronic insomnia in adults and includes stimulus control, sleep restriction, and cognitive therapy [35]. A mental health clinician noted that patients were unable to keep sleep diaries (a component of CBT-I) due to chaotic environments, and that many patients do not have a phone compatible with CBT-I mobile applications (Provider 1). A peer specialist noted being only able to successfully teach bedtime relaxation techniques (another approach of CBT-I) to veterans once they had received permanent housing (Provider 6).

About half of the providers, including primary care and mental health providers and peer specialists, commented on the use of medications for sleep. Several expressed concerns that homeless-experienced veterans feel groggy with use of medications, while others noted that these medications might be poor options for patients with substance use disorders, given risk of misuse. Providers also noted the potential for loss or theft of these medications. A peer specialist noted that in some temporary housing settings, medications are handed out by a non-medical staff member who does not know indications for medications. In this case, when the veterans are not reminded that they have a sleep medication, they often forget to take it (Provider 9). 

Most providers commented on the difficulty of treating OSA within this population. In particular, they expressed concern about patients’ use of positive airway pressure (PAP) machines, which are the most commonly recommended treatment. Two providers noted that PAP machines need to be plugged into an electrical outlet throughout the night, which may be unavailable to those experiencing homelessness. A nurse noted that these machines are difficult to carry and keep organized (Provider 13). One provider pointed out that in sleep settings with shared rooms or congregate living, PAP machines are unwelcome because roommates find them noisy (Provider 4). 

### 3.4. Organizational Factors

#### 3.4.1. Sleep Clinic Logistics

Clinic logistics were identified as barriers to sleep disorder treatment by primary care providers, mental health providers, and peer specialists. Several noted that appointments for sleep treatment are burdensome. They described an onerous process in which patients need a phone to set up their sleep appointment and may be required to return to clinic multiple times for sleep studies, equipment pick-up, and device malfunctions. A peer specialist mentioned that it is also difficult for veterans to get transportation to sleep-related classes at the medical center (Provider 3).

#### 3.4.2. Competing Structural Determinants

Nearly half of providers, including all the mental health and two primary care providers, brought up competing life priorities as a barrier to addressing sleep problems. They noted that priorities like finding housing, working, and providing for children often take precedence over sleep. A mental health clinician explained that patients often prioritize work over sleep when they work night shifts or have long commutes (Provider 10). A primary care provider noted that few patients will complete a sleep disorders consult appointment before they have housing (Provider 5). One provider reported that patients feel that they can just “tough it out” when it comes to poor sleep (Provider 10). 

#### 3.4.3. Frequent Transitions

Transitions—for example, from the street, to a shelter, to permanent housing—were noted to impact sleep. A peer specialist described the experience of moving from homelessness to new housing as disconcerting; veterans often find their new homes to be too quiet and unfamiliar, which impacts their sleep (Provider 3). A nurse observed that her patients would leave the television on all night, perhaps to mimic the experience of sleeping in a louder setting (Provider 13).

## 4. Discussion

This qualitative inquiry is the first to our knowledge to specifically explore insufficient sleep among homeless-experienced veterans. Although there has been increasing attention to the disproportionate impact of poor sleep on under-resourced populations, little has been explored among homeless-experienced veterans despite efforts to improve housing and chronic disease outcomes. 

Our results demonstrated that sleep is a significant issue among the homeless-experienced veteran population that can affect mental and physical health along with quality of life. The findings build upon a modest prior literature exploring sleep problems among the general homeless population and can provide specific implications on multiple levels—individual, interpersonal, environmental, organizational, and on a policy level—for improving care for veterans experiencing homelessness and sleep disturbance and inform future research priorities. 

### 4.1. Individual Level

Insufficient and low-quality sleep are known to decrease mental well-being among people experiencing homelessness [36]. We found poor sleep in this veteran population is often tied to mental illness and substance use, problems that are increased among homeless-experienced veterans [37]. Given the sleep problems among homeless-experienced veterans described in this study and among non-veterans described in prior studies [13,14,15,16,17], and the growing awareness of addressing sleep disparities, healthcare providers should consider screening homeless-experienced veterans for sleep problems. Due to the vast differences in sleep settings between homeless and housed veterans, it would be appropriate to adapt screening measures—such as the Pittsburgh Sleep Quality Index [38]—specifically for this population. This tool, which has been used among structurally vulnerable groups including those with mental illness [39], already includes items questioning whether some aspects of the environment disrupt sleep (including temperature and disruptive roommates) and whether sleep medications are used. An adaptation for homeless-experienced veterans might inquire about other issues highlighted in our interviews, such as substance use, nightmares, feeling unsafe, and needing to leave shelters early in the morning. An adapted screening tool would allow clinicians to better inquire about, detect, understand, and address sleep problems within this population. Further, providers working with homeless-experienced veterans should familiarize themselves with potential factors affecting sleep across different shelter environments, such as crowdedness and inflexible schedules in emergency shelters, and noise issues in transitional housing. Through using screening tools and opening discussions about potential environmental and structural factors impacting sleep instead of focusing only on the symptoms of poor sleep, providers can help empower veterans to bring up these issues that may otherwise go undetected. 

Providers in our study pointed out the significant issue that sleep problems among veterans sleeping on the street tend to persist in other settings, including permanent supportive housing. Our findings are supported by the civilian literature, with findings that insufficient sleep persists among civilians even after moving into stable housing [13,40]. One study found that 28% of homeless-experienced individuals living in permanent supportive housing programs had moderate or severe sleep disturbance, which was associated with impairment in activities of daily living and pain [41]. Additionally, providers specifically mentioned that mental health problems can remain unresolved after obtaining secure housing and continue to negatively impact sleep. These findings demonstrate that on an individual level, community health providers and homeless service providers such as case managers, should be sensitive to mental health problems, limited functioning, pain, and sleep problems as people transition into housing, and that these problems may not resolve immediately with housing. This also points to a need for research exploring how interventions can improve sleep after veterans obtain permanent housing, including the impact on chronic health conditions and long-term outcomes. 

### 4.2. Interpersonal Level

The main interpersonal concern cited by providers was safety when sleeping, which was often discussed as veterans staying awake at night to protect themselves. Our findings align with safety concerns that are documented among civilians experiencing homelessness, particularly relating to fears of staying in shelters where they are concerned there is a risk of assault [42]. Ultimately, providing veterans permanent housing is necessary to address these concerns. However, in the short term and if adequate permanent housing is not available, agencies should consider increasing safety measures, such as locks for belongings, to address concerns about safety while sleeping in shelters. Tent communities and safe parking areas with access to medical and mental health services, which are being piloted in cities such as Los Angeles and San Francisco, could help some veterans who may not feel comfortable staying in shelters while awaiting permanent housing [43,44,45]. The West Los Angeles VA is also supporting a street medicine program within an encampment site on-campus [46]. In fact, a survey of people experiencing homelessness in San Francisco found that most participants preferred a legal tent site to a shelter [47]. However, more research is needed to understand the impact of these safe parking and tent community programs on sleep and health outcomes. Community health and homeless services providers can ask patients about safety concerns and their impact on sleep and mental health. They can also advocate for permanent housing and short-term solutions of enhancing safety among veterans experiencing homelessness that align with the voiced needs of veterans, such as safe tent communities. 

We also found that veterans experiencing homelessness are also often kept awake by loud noises and awakened early because of shelter schedules. Shelters have been documented as often having “stringent” and “complex” rules, including curfews and mandatory meetings, and violation of these rules can result in discharge from the shelter [48]. Shelters and housing facilities should consider loosening rules that are poorly conducive to good sleep, such as rigid sleep hours. Other environmental changes and policies to enhance sleep include offering white noise machines or earplugs and separating clients who work at night. 

### 4.3. Environmental Level

We found that homeless-experienced veterans lose sleep due to a lack of control over their environment. This echoes a recent qualitative analysis among non-veteran individuals with homeless experiences, which found that participants in both sheltered and unsheltered settings reported lost sleep due to proximity to strangers, adverse weather conditions, hard surfaces, and noise [49]. 

We also found that the environment impacts the use of sleep disorder treatments. Despite living in a vastly different environment, veterans experiencing homelessness are often offered the same clinical interventions for sleep problems as patients who are housed. Regarding OSA treatment, PAP use is already known to be problematic in some under-resourced populations; for example, people with lower socioeconomic status are less likely to be receptive to PAP use, often due to costs of treatment [50]. Mental illness is also a factor; for instance, veterans with PTSD have higher levels of PAP-related anxiety, and PAP-anxiety predicts non-adherence [51]. Our study found that within the homeless population, the environment can limit use of PAP machines due to limited electrical outlets, storage options, and clean water for use and cleaning of these devices. Providers can consider alternatives to PAP therapy, such as use of oral appliances for OSA in patients unable to use PAP [52]. Shelters might also consider grouping clients with PAP machines by electrical outlets and in independent rooms so as not to disturb others’ sleep. PAP non-adherence is important for chronic disease management—untreated OSA can lead to poor health outcomes like cardiovascular disease, stroke, and metabolic disease. It can also lead to outcomes like poor functioning at work, daytime sleepiness, and car accidents [53]. Addressing PAP access and usability in this population could have lasting impacts. 

Our study also illuminated the limitations of CBT-I among homeless-experienced veterans. CBT-I, which is first-line therapy for chronic insomnia, has demonstrated good outcomes for veterans, including those with mental illness [54]. Unfortunately, providers described that chaotic environments while homeless make it very difficult to use relaxation techniques before bed. These environments are also not conducive to keeping sleep diaries and regular schedules as is recommended in CBT-I. Additionally, mobile apps are regularly used in CBT-I treatment, and access to these is unreliable for those experiencing homelessness without regular availability of outlets. 

These barriers compound the well-described barriers to CBT-I within the housed population, such as travel requirements, costs, and prolonged engagement with practitioners [55]—which may be especially salient in under-resourced populations due to work, childcare responsibilities, and limited transport. Despite the barriers in receiving CBT-I treatment, the VA has a commitment to providing CBT to veterans with chronic homelessness (CBT-H) [56]. Efforts should be made to make CBT-I (in addition to general CBT) more readily available and tailored to the veteran homeless population. For instance, patients’ sleep may benefit most from reducing substance use, identifying times that are safe for sleep, and creating other strategies to maximize sleep given their environmental limitations. 

Community healthcare agencies could also improve access to CBT-I through telehealth. Telehealth services have rapidly gained relevance during the COVID-19 pandemic [57]; telehealth could also be used to bridge more long-standing barriers like limited transportation and childcare services. The VA offers programs that connect veterans to internet services and personal electronic tablets for those with limited tele-capabilities [58]. Barriers could remain, yet emerging research during the pandemic has shown steady and growing access to mental health care through the use of telehealth [59]. 

### 4.4. Organizational Level

The organizational factors impacting sleep among veterans experiencing homelessness include burdensome sleep clinic appointments, difficulty with transportation, telephone confirmations of appointments, and outpatient sleep studies in precarious environments. While some of these barriers have been noted in the general veteran population [60], they are likely amplified in the homeless-experienced veteran population due to lack of transportation, very poor sleep environments, poor access to phone charging, and competing priorities. Sleep clinic providers should consider modifications to address these problems, such as increasing availability of walk-in appointments and expanding capacity for overnight inpatient sleep studies for veterans who are homeless. Community health agencies can assist with transportation and appointment scheduling when these services are available. One prior qualitative analysis found that people experiencing homelessness described difficulty concentrating, completing tasks, and interacting with people due to poor sleep. They wondered whether these sleep-related problems could be contributing to their inability to secure housing [49]. We therefore do not recommend that providers hold off on sleep treatment until veterans are housed and instead tailor the services to the needs of veterans experiencing homelessness. 

### 4.5. Policy Level

Finally, at the policy level, many cities have policies and ordinances that actively disrupt sleep among people experiencing homelessness, such as limiting areas that people can safely sleep, or conducting city sweeps or cleanups that may limit the ability to carry PAP machines (as well as water to use with PAP humidifiers) to new locations. One example is the requirement that tents be dissembled early in the morning in some cities [19]. Although these policies were relaxed during COVID-19 due to CDC recommendations in many localities [61], these policies should be reconsidered following the pandemic. Allowing people to sleep safely in tents and stopping city sweeps that lead to throwing out PAP supplies and water, would likely lead to increased sleep, and therefore improve health and functioning within this structurally vulnerable population. 

Efforts are also needed to increase access to safe and affordable permanent housing for veterans to start to address the barriers we identified to sleep and sleep treatment, including poor environmental conditions, lack of safety, and competing priorities [62,63,64]. Even once housed, providers cautioned that sleep does not always improve. Further research is needed to determine the relationships between histories of homelessness, permanent supportive housing, sleep, chronic health, and mental health outcomes. Longer-term sleep intervention studies are needed in the recently housed population. Providers caring for this population can use their expertise to advocate for policies that increase access to permanent supportive housing and against policies that worsen sleep. 

### 4.6. Limitations

This project involved interviews with providers of homeless-experienced veterans and was conducted at one VA medical center through convenience sampling. The study was limited by a relatively small sample size. All providers practiced within one VA healthcare system, which may limit generalizability. Additionally, most participants were clinicians; this excludes perspectives of other service sectors working with veterans experiencing homelessness, such as providers in homeless shelters in the community. 

A main limitation was that the interviews were conducted with providers who work with homeless-experienced veterans rather than homeless-experienced veterans themselves. Although the providers all worked closely with homeless-experienced veterans and provided important perspectives on this topic, studies centering the experiences and perspectives of homeless-experienced veterans are also needed. Future studies should explore experiences related to sleep voiced by homeless-experienced veterans themselves, especially populations known to face increased stressors during homelessness such as veterans who identify as women, and/or as lesbian, gay, bisexual, transgender, or queer (LGBTQ+), and minoritized veterans. We did not specifically ask about differences in sleep experiences among genders, and this may be particularly helpful to explore for veterans who identify as women—who may experience heightened concerns about safety and environment pertaining to sleep. Future work can build upon the themes identified in this study to obtain additional perspectives and design tailored sleep-related interventions for homeless-experienced veterans. However, despite these limitations, these findings highlighting the sleep issues that homeless-experienced veterans often endure—as voiced by providers with expertise in caring for them—can be useful for informing clinical, social, and structural changes to lessen the impact of sleep deprivation on homeless-experienced veterans. 

## 5. Conclusions

We found that providers in various roles who care for homeless-experienced veterans identified a range of factors contributing to sleep problems among these individuals, occurring at multiple levels of the social-ecological framework. These findings suggest opportunities for community providers, healthcare systems, and homeless services to improve care in this population, including screening for sleep problems through adapting screening tools, implementing interventions to address poor sleep after veterans obtain housing, addressing safety concerns related to sleep, tailoring CBT-I for this population, and addressing clinical barriers, among others. Our findings also support the need for future interventions to address sleep disparities among veterans experiencing homelessness.

## Data Availability

Author elects not to share data: These findings are from qualitative data; while de-identified, the nature of the data does not ensure complete participant anonymity. Participants did not give permission for their data to be available publicly for other studies.
